# All-electrical reading and writing of spin chirality

**DOI:** 10.1126/sciadv.add6984

**Published:** 2022-12-14

**Authors:** Fan Li, Yicheng Guan, Peng Wang, Zhong Wang, Chi Fang, Ke Gu, Stuart S. P. Parkin

**Affiliations:** NISE Department, Max Planck Institute of Microstructure Physics, Halle, Germany.

## Abstract

Spintronics promises potential data encoding and computing technologies. Spin chirality plays a very important role in the properties of many topological and noncollinear magnetic materials. Here, we propose the all-electrical detection and manipulation of spin chirality in insulating chiral antiferromagnets. We demonstrate that the spin chirality in insulating epitaxial films of TbMnO_3_ can be read electrically via the spin Seebeck effect and can be switched by electric fields via the multiferroic coupling of the spin chirality to the ferroelectric polarization. Moreover, multivalued states of the spin chirality can be realized by the combined application of electric and magnetic fields. Our results are a path toward next-generation, low-energy consumption memory and logic devices that rely on spin chirality.

## Teaser

Spin chirality can be electrically detected and manipulated at the same time, with applications for memory and computing devices.

## INTRODUCTION

Spin chirality, formed by noncollinear or noncoplanar arrangements of spins, is of great research interest owing to the rich variety of physics that it can engender, including Berry phase (*1*), chiral spin liquid states (*2*), giant anomalous Hall and anomalous Nernst effects (ANEs), as well as various unconventional spin transport phenomena (*3*–*8*). As a central concept, vector spin chirality (**S**_1_** × S**_2_) and scalar spin chirality [**S**_1_·(**S**_2_ × **S**_3_)] are defined to describe the alignment of spins at adjacent sites (**S**_*i*_) for noncollinear and noncoplanar cases, respectively. The spin chirality can, correspondingly, be switched in direction or sign, leading to diverse chirality-based novel physics. The switching of the spin chirality can play a similar role to that of the “0” and “1” states of bits in digital information technologies and, thus, has potential for next-generation memory and logic devices (*9*, *10*). Consequently, the reading and writing of the spin chirality is a topic of considerable interest but has remained elusive to date.

In general, spin chirality can be read electrically by various spin transport phenomena, as recently demonstrated in metallic chiral antiferromagnets (*11*–*14*). In these materials, the current-induced manipulation of the antiferromagnetic (AFM) state has recently been realized but the spin chirality remains unchanged during the switching process (*15*–*17*). Direct switching of the spin chirality calls for new strategies and materials such as multiferroic insulating oxides. The electric field switching of the spin chirality in such systems has been demonstrated through the coupling between the chiral AFM order and the ferroelectric (FE) polarization (*18*–*20*). However, the electrical detection of the spin chirality in these insulating systems has not yet been demonstrated because of the lack of conduction electrons, which is circumvented by the use of nonelectrical methods such as x-ray dichroism, neutron scattering, and optical second harmonic generation (*21*–*23*). Here, we propose a method to realize all-electrical reading and writing of spin chirality in a multiferroic insulator. The electrical reading is achieved by using the spin Seebeck effect (SSE), which does not depend on conduction electrons but rather on magnon transport that is influenced by the spin chirality, while the writing is via electric field manipulation of the FE polarization.

## RESULTS

### Concept of all-electrical reading and writing of spin chirality

**Fig. 1.**
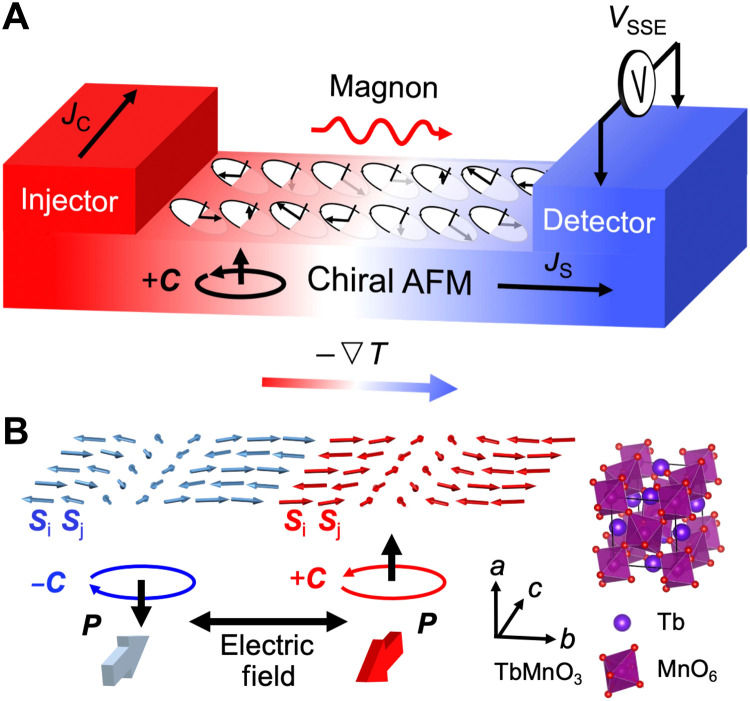
All-electrical reading and writing of spin chirality. (**A**) Illustration of electrical readout of the spin chirality via the SSE in a chiral AFM insulator. An electrical current (*J*_C_) in the spin injector generates a temperature gradient (∇*T*) in the chiral AFM insulator. Thus, a magnon spin current (*J*_S_) forms due to the coupling between the spin chirality (**C**) and the out-of-plane spin precession component of the left- and right-handed magnons. Subsequently, spin Seebeck voltage (*V*_SSE_) is read out via the inverse spin Hall effect by the spin detector of a heavy metal. By measuring *V*_SSE_, the spin chirality in the AFM insulator can be detected. (**B**) Schematic diagram of electric field switching of the out-of-plane spin chirality ( **C**) via switching of the in-plane FE polarization (**P**) for multiferroic TbMnO_3_. The spin chirality (**C* = *S***_i_* × **S***_j_*) is formed by the adjacent spins (**S***_i_*, **S***_j_*) along the *b* axis. **C** and **P** are along the *a* and *c* axes of the TbMnO_3_ lattice, respectively.

The SSE refers to the generation of a spin “voltage” induced by a temperature gradient (*24*). Arising from magnon spin currents in magnetic insulators, the SSE has been observed in both ferromagnetic and collinear AFM insulators, although there is no conduction-electron spin current (*25*–*29*). Here, we extend the observation of the SSE to a chiral AFM insulator and use it for the electrical detection of the spin chirality. A nonlocal geometry is adopted for measurement of the SSE, as sketched in Fig. 1A. When a charge current (*J*_C_) flows through the spin injector (a heavy metal nanostrip) on top of an AFM insulator, the joule heating induces a temperature gradient (∇*T*). Because of the coupling between magnons and the spin chirality (**C**) in the chiral AFM layer, an imbalance in the population of the left- and right-handed magnons appears. The right-handed magnons are favored by the spin chirality upward (+**C**), because the partial spin precession out of plane has the same chirality as that of the AFM spin spiral. Thus, a magnon spin current (*J*_S_) forms under the temperature gradient and can be read out in the spin detector via the inverse spin Hall effect. In this way, a spin Seebeck voltage (*V*_SSE_) related to the spin chirality is generated, providing a method for electrical readout of the spin chirality in insulating systems.

Subsequently, taking the required geometrical conditions into account, we integrate the electrical readout via the SSE with electric field writing of the spin chirality in multiferroic systems. Here, TbMnO_3_ is used as an example. As sketched in Fig. 1B, this material is a multiferroic insulator that displays an incommensurate AFM order with a spin spiral along the *b* axis below its magnetic ordering temperature, *T*_N_ = 41 K. A vector spin chirality oriented along the *a* axis is determined by the alignment of adjacent spins, i.e., **C** ***= *S**_*i*_ × **S**_*j*_, and is coupled to an FE polarization (**P**) along the *c* axis below 28 K (*30*–*32*). Considering the geometrical setup for detection and manipulation of the spin chirality, an out-of-plane alignment of the spin chirality for the detection via the SSE and an in-plane FE polarization for the simultaneous electric field control are adopted, which can be experimentally satisfied by the epitaxial growth of TbMnO_3_ (100). In this way, by integrating the SSE with the multiferroic coupling in a suitable geometry, all-electrical reading and writing of spin chirality can be realized.

### Electrical reading of the spin chirality in TbMnO_3_

**Fig. 2.**
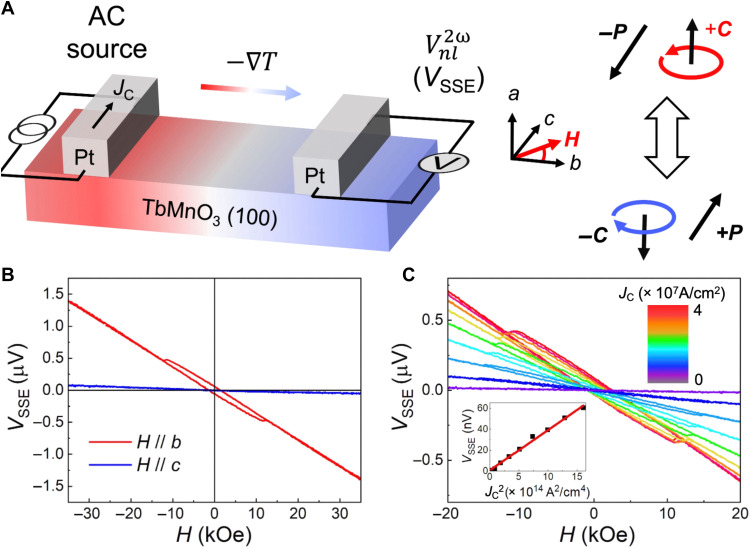
Electrical readout of the spin chirality in TbMnO_3_. (**A**) Sketch of the nonlocal measurement of the SSE in TbMnO_3_. In devices fabricated from TbMnO_3_ (100) epitaxial films, the spin chirality is aligned out of plane (**C** // *a*). Pt nanostrips as a spin injector and a spin detector are oriented parallel to the FE polarization (**P** // *c*). An AC source is connected to the spin injector, and *V*_SSE_ is read out from a second harmonic voltage ($\$V_{nl}^{2\omega }\$$) from the spin detector. A magnetic field (*H*) is applied in the *b*-*c* plane. (**B**) Magnetic field–dependent *V*_SSE_ measured at 4 K for *H* // *b* and *H* // *c*. A clear hysteresis loop is observed for *H* // *b*, but there is no signal for *H* // *c*. (**C**) Influence of the current density (*J*_C_) on the magnetic field dependence of *V*_SSE_ at 4 K for *H* // *b*. Dependence of *V*_SSE_(*H* = 0) on *J*_C_^2^ is plotted in the inset, which exhibits a linear relationship.

To detect the spin chirality via the SSE, a (100)-oriented TbMnO_3_ thin film is grown on LaAlO_3_ (110) with an epitaxial relationship of TbMnO_3_ (100) [001] // LaAlO_3_ (110) [001], owing to the small in-plane lattice misfit between TbMnO_3_ and LaAlO_3_ (as shown in table S1). The TbMnO_3_ (100) thin film is of high quality (see figs. S1 to S3) and is fabricated into nonlocal devices for the SSE measurements. As depicted in Fig. 2A, the spin chirality **C** in TbMnO_3_ (100) is oriented along the out-of-plane *a* axis, while Pt nanostrips along the in-plane direction of the FE polarization **P** (the *c* axis) are fabricated, satisfying the geometry for all-electrical reading and writing of spin chirality as mentioned above. With an alternating current (AC) source connected to the Pt spin injector, *V*_SSE_ can be read out by measuring the second harmonic voltages ($\$V_{nl}^{2\omega }\$$) from the Pt spin detector. Details are discussed in Materials and Methods. In addition, the alignment of the Pt strips along the *c* axis, that is, the axis of FE polarization, enables the subsequent electric field switching of the spin chirality via control of the FE polarization (Fig. 2A, right), promising the electrical writing of the spin chirality.

Considering that the spin spiral in TbMnO_3_ can be easily modulated along the *b* axis (the in-plane spin spiral axis) (*33*), the responses of the SSE to the magnetic field along the *b* axis is first examined, as shown in Fig. 2B. The field-dependent *V*_SSE_ shows a clear hysteresis loop, indicating a magnetic reversal of the spin spirals. The linear background is ascribed to the magnetic modulation of the spin spirals (as discussed further in fig. S6). As the spin spirals are reversed, an opposite vector spin chirality is attained. Here, the ANE of TbMnO_3_ and the ANE due to the proximity-induced magnetic moment in Pt can be ignored, owing to the insulating and AFM nature of TbMnO_3_, respectively. In comparison, for *H* // *c*, *V*_SSE_ remains almost unchanged, which is ascribed to the angular dependence of the SSE due to the requirement of spin polarization with a specific direction (fig. S4). Thus, by confirming that the reversal of the spin chirality can read out via the SSE with a magnetic field applied along the *b* axis, subsequent SSE measurements are carried out with *H* // *b*.

Various current densities (*J*_C_) are applied to confirm that the measured voltage originates from the SSE, given that a larger *J*_C_ would result in a larger temperature gradient. As illustrated in Fig. 2C, an enlarged hysteresis loop is observed as *J*_C_ increases. Moreover, a linear dependence of *V*_SSE_(*H* = 0) on *J*_C_^2^ is demonstrated in the inset, further verifying the dominant role of the SSE in the measured magnetic field–dependent voltage, considering the thermal nature of the SSE. Consequently, we find that the reversal of the out-of-plane spin chirality via a magnetic field applied along the spin spiral axis can be electrically detected by the SSE.

### Integration of electric field writing of the spin chirality with reading in TbMnO_3_

**Fig. 3.**
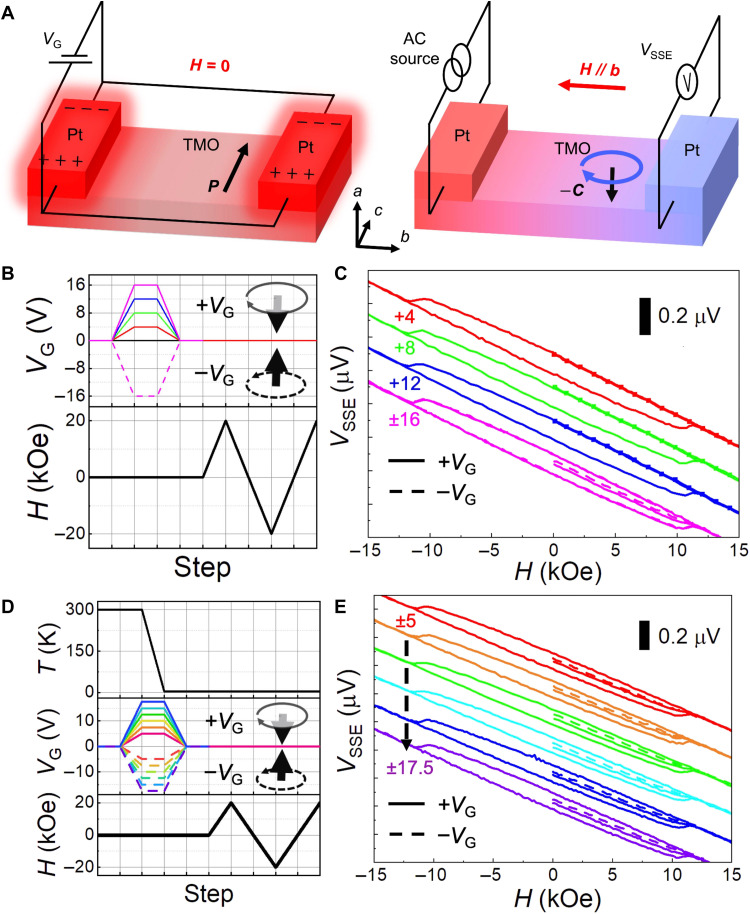
In situ electric field switching of the spin chirality at 4 K. (**A**) Schematic diagram showing gating with *V*_G_ and measurement of *V*_SSE_. *V*_G_ is applied to two Pt strips (left panel) so as to generate an in-plane electric field along the *c* axis, resulting in thermally assisted switching of the FE polarization **P** and the coupled spin chirality **C**. *V*_SSE_ is then read out after *V*_G_ is set to zero (right panel). (**B**) Variation of *V*_G_ and *H* during the gating process and subsequent measurements. Insets show how **C** depends on *V*_G_. (**C**) *V*_SSE_ at *H* = 0 and subsequent hysteresis loops after application of various *V*_G_. The initial magnetization process of *V*_SSE_ from *H =* 0 is indicated by square symbols for *V*_G_ = +4, +8, and +12 V and by solid and dashed lines for *V*_G_ = +16 and −16 V. Two distinct values of the initial *V*_SSE_(*H* = 0) are observed when *V*_G_ is increased to ±16 V. (**D**) Temporal variation of *T*, *V*_G_, and *H* during an initial zero–magnetic field cooling for various *V*_G_ and subsequent measurements. (**E**) Initialization of the spin chirality by cooling from 300 K in various ±*V*_G_. Two different initial *V*_SSE_(*H* = 0) values are achieved by cooling in ±*V*_G_, for a wide range of |*V*_G_| varying from 5 to 17.5 V, indicating that *V*_SSE_(*H* = 0) is always smaller for the downward spin chirality (−**C**) obtained for +*V*_G_.

The multiferroic properties of TbMnO_3_ facilitate the in situ electrical switching of the spin chirality through the coupled FE polarization. Therefore, we next focus on the integration of the in situ electric field switching of the spin chirality with the electrical reading via the SSE. As illustrated in the left panel of Fig. 3A, a gate voltage (*V*_G_) is first applied to both of the Pt strips oriented along the *c* axis with *H* = 0 , leading to a thermal-assisted switching of the FE polarization together with the reversal of the spin chirality. As follows, *V*_G_ is set to zero and *V*_SSE_ is read out with the nonlocal setup to detect the variation of the spin chirality (Fig. 3A, right). Details can be found in Materials and Methods.

First, we discuss the role of in situ gating effect on *V*_SSE_ at 4 K. The variations of *V*_G_ and *H* are schematically illustrated in Fig. 3B, where *V*_G_ is first set to +4, +8, +12, and ±16 V for gating before the measurement of *H*-dependent *V*_SSE_ with *H* increasing from 0. As shown in Fig. 3C, when *V*_G_ increases from +4 to +12 V, the initial magnetization process from *V*_SSE_(*H* = 0) remains unchanged, as shown by the lines with square symbols, indicating that there is no variation of the spin chirality. Notably, when |*V*_G_| increases to 16 V, the scenario changes: *V*_SSE_(*H* = 0) decreases to two distinct values between the two zero–magnetic field values of the hysteresis loop, as shown by the solid and dashed magenta lines, respectively. For ±*V*_G_ of the same magnitude, the heating effect is identical, but the electric fields are opposite. Thus, the decrease of *V*_SSE_(*H* = 0) from the hysteresis loop can be ascribed to the thermal demagnetization of the net magnetic moment induced by the spin-flop transition, while the difference between the two *V*_SSE_(*H* = 0) values is due to the opposite contributions of the spin chirality, which is reversed by the electric field. In this view, the reversal of the spin chirality induced by ±*V*_G_ can be electrically detected by the variation of SSE when |*V*_G_| reaches 16 V.

Electrical switching of the spin chirality can also be realized by manipulating the initial FE polarization through cooling from room temperature with ±*V*_G_. Zero–magnetic field cooling with various *V*_G_ is performed, and the experimental steps are illustrated in Fig. 3D. In general, *V*_G_ is first applied and the system is cooled down to 4 K with *H* = 0. Then, *V*_G_ is set to zero by 0.1 V/s, and subsequently, *H*-dependent *V*_SSE_ is measured with *H* increasing from zero. Because the curie temperature of the FE polarization in TbMnO_3_ is 28 K, an electric field cooling process from 300 to 4 K, which crosses the paraelectric-to-FE phase transition, can lower the required *V*_G_ for switching the spin chirality. As depicted by the solid and dashed lines in Fig. 3E, two distinct *V*_SSE_(*H* = 0) values related to the opposite spin chirality are achieved by cooling with +*V*_G_ and −*V*_G_, respectively, where *V*_G_ varies over a wide range from 5 to 17.5 V. Compared to the condition without zero–magnetic field cooling process, where the required |*V*_G_| value for the reversal of the spin chirality is 16 V, the present cooling process across the curie temperature of FE order greatly decreases the required |*V*_G_| value. This is remarkable as it demonstrates that, by taking advantage of the electric field switching of the spin chirality through the FE polarization, electrical writing of the spin chirality is realized together with the electrical readout of the spin chirality through SSE in the AFM insulator.

### Multivalued states via combined electrical switching and magnetic manipulation

**Fig. 4.**
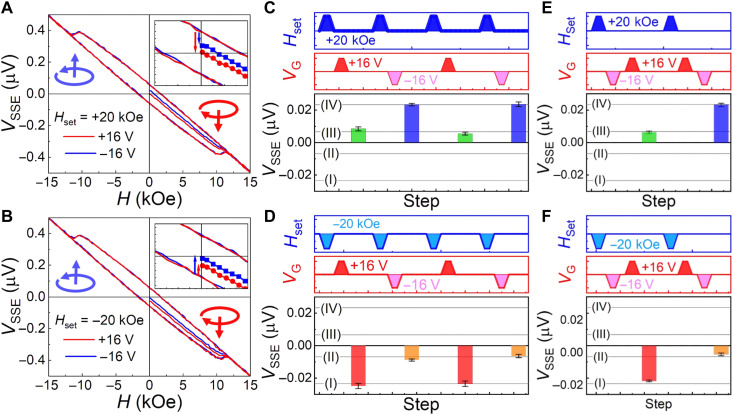
Multivalued states achieved by control of the spin chirality. (**A** and **B**) *V*_G_ setting of *V*_SSE_(*H* = 0) states after the application of a magnetic field (*H*_set_) of +20 and −20 kOe, respectively. Different *V*_SSE_(*H* = 0) values are attained for opposite *H*_set_, and a lower *V*_SSE_(*H* = 0) is always observed for +*V*_G_ with the same *H*_set_ when compared to the case of −*V*_G_, as marked by the arrows in the insets. Spin chirality with a downward and upward arrow is depicted, corresponding to the application of +*V*_G_ and −*V*_G_. (**C** and **D**) Reversible and reproducible control of *V*_SSE_ under repetitive applications of *H*_set_ and *V*_G_. The top two panels depict the application process of *H*_set_ and *V*_G_, while the bottom panel shows the multivalued *V*_SSE_ measured after setting *H*_set_ and *V*_G_ to zero. Four values of *V*_SSE_ indicated by (I) to (IV) are attained via the magnetic and electric field control of the spin chirality. (**E** and **F**) Switching of *V*_SSE_ via the application of ±*V*_G_ under a fixed *H*_set_. When *H*_set_ is fixed, a direct application of an opposite *V*_G_ leads to a limited variation of *V*_SSE_, that is, for *H*_set_ = +20 kOe, *V*_SSE_ is limited to (III) and (IV), while for *H*_set_ = −20 kOe, *V*_SSE_ is limited to (I) and (II).

Beyond the electric field switching of the spin chirality, we note that the spin chirality can be further manipulated by the magnetic field–induced modulation of the net magnetic moment via spin-flop transition. In this case, by using the magnetic manipulation together with the electric field switching of the spin chirality, multivalued states in addition to "0" and "1" can be attained. Experimentally, with the temperature kept at 4 K, magnetic fields (*H*_set_) of ±20 kOe are applied and then set to zero before electric field switching with *V*_G_ = ±16 V, to introduce a variation in *V*_SSE_(*H* = 0). Subsequently, *V*_SSE_ is measured in the absence of both *V*_G_ and *H*_set_. Figure 4A shows the initial *V*_SSE_(*H* = 0) and its variation under the combined applications of +*H*_set_ and ±*V*_G_ at 4 K. The two *V*_SSE_(*H* = 0) states set by ±*V*_G_ are asymmetric with respect to the origin, as exhibited by the arrows in the inset. This is distinct from the states attained by electric field cooling with ±*V*_G_ in zero magnetic field (Fig. 3C). This indicates an additional role of *H*_set_ in the modulation of the spin chirality. Furthermore, by using −*H*_set_, another two distinct *V*_SSE_ values are further realized, as shown in Fig. 4B. Thus, multivalued states can be realized by combined applications of *H*_set_ and *V*_G_. Nevertheless, a smaller *V*_SSE_(*H* = 0) is always observed in the case of +*V*_G_, for both +*H*_set_ and −*H*_set_, as indicated by the red and blue arrows in the insets, indicating the dominant role of *V*_G_ in switching the spin chirality.

Next, we measure *V*_SSE_(*H* = 0) values after applying a series of sets of values of *H*_set_ and *V*_G_ to explore the reproducibility and reversibility of the multivalued states. Considering the small error bars (estimated from fig. S7), the four *V*_SSE_ states denoted by (I) to (IV) can be readily distinguished from each other by the electrical readout; moreover, they are reversible and reproducible after the repeated applications of *H*_set_ and *V*_G_, as shown in Fig. 4 (C and D). Once the sign of *H*_set_ is determined, the *V*_G_-controlled states are limited to two values and can be reversibly switched by directly applying opposite *V*_G_ (Fig. 4, E and F). Thus, we find that, with additional magnetic manipulation of the spin chirality, the electric field switching of the spin chirality is able to induce multivalued *V*_SSE_ states with good reproducibility.

### Contribution of the reversed spin chirality to SSE

**Fig. 5.**
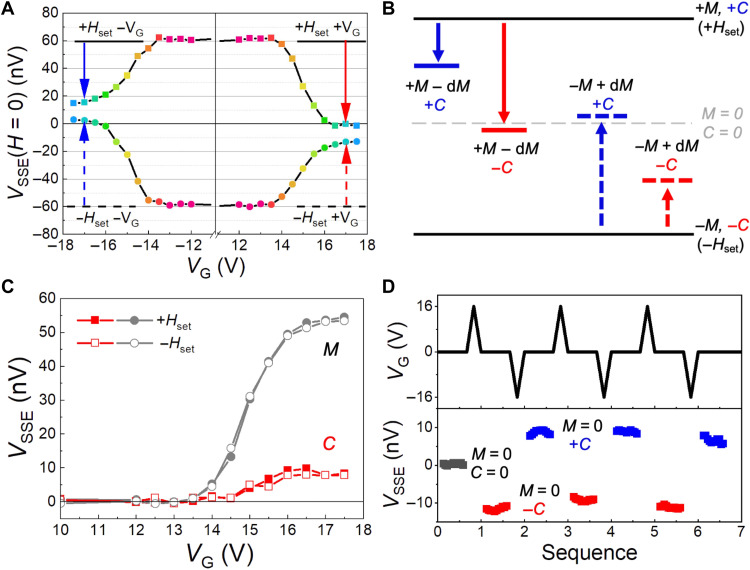
Thermal-assisted switching of spin chirality for multivalued states. (**A**) Dependence of *V*_SSE_(*H* = 0) on *V*_G_ at 4 K, after initial magnetization via *H*_set_ = ±20 kOe. The asymmetry of the curves for ±*V*_G_ demonstrates the contribution of **C** to *V*_SSE_. (**B**) Schematic diagram of the gating-induced variation of *V*_SSE_ due to the variation of **C** and **M**. (**C**) Contributions of **C** and **M** to the variation of *V*_SSE_ as a function of *V*_G_. Lines with solid and open symbols denote the cases of initialization with +*H*_set_ and *−H*_set_, respectively. (**D**) Reversible and reproducible control of *V*_SSE_(*H* = 0) via electric field switching of **C** in the case of **M** = 0, which is achieved by zero–magnetic field cooling from 300 K. The top panel illustrates the application of *V*_G_, while the bottom panel plots the corresponding variation of *V*_SSE_(*H* = 0) measured with *V*_G_ = 0. After gating with *V*_G_ = +16 V (−16 V), the spin chirality of −**C**** (+**C**) is attained, leading to a negative (positive) *V*_SSE_, compared to the initial status of *V*_SSE_ = 0 due to complete demagnetization (**M** = 0, **C** = 0).

The multivalued states are achieved by magnetic manipulation of the spin chirality, which can be attributed to an additional magnetic moment via the spin-flop transition. Therefore, a further interpretation about the contribution of the spin chirality (**C**) and the magnetic moment (**M**) to the SSE is of great interest for a deep understanding of the multivalued states. To reveal the contribution of **C** and **M** to the SSE, *V*_SSE_(*H* = 0) at 4 K is measured after initializing the magnetization with *H*_set_ = +20 and −20 kOe and gating with various ±*V*_G_ under *H* = 0. As shown in Fig. 5A, when |*V*_G_| is below 13.5 V, *V*_SSE_(*H* = 0) remains the same, indicating that *V*_G_ is not large enough to induce either a thermal demagnetization or an electric field reversal of the spin chirality. When |*V*_G_| is larger than 13.5 V, a decrease of |*V*_SSE_(*H* = 0)| occurs due to a thermal demagnetization. A larger *V*_SSE_(*H* = 0) is always achieved by *−V*_G_, corresponding to a spin chirality of +**C**, when compared to the case of +*V*_G_ of the same magnitude. The asymmetric curve for ±*V*_G_ indicates that the spin chirality reversed by the electric field leads to a remarkable difference in the SSE, distinct with the same variation of the SSE due to the identical thermal demagnetization.

The contributions of **C** and **M** to the variation of the SSE are further sketched in Fig. 5B. Two initial states (+**M**, +**C**) and (*−***M****, *−***C****) are achieved by +*H*_set_ and *−H*_set_, respectively, corresponding to the maximum and minimum values of *V*_SSE_ in Fig. 5A. Because of the demagnetization (−d**M**) and the reversal of the spin chirality (+**C** ↔ −**C****) in the gating process, additional four states can be attained for ±*V*_G_ of the same magnitude, as marked by the four arrows, accounting for the observation of the multivalued states. Furthermore, the contribution of **C** and **M** under different *V*_G_ values can be quantitatively estimated (details about the estimation can be found in Materials and Methods), as displayed in Fig. 5C. With *V*_G_ increasing until 13.5 V, the contributions of **C** and **M** to *V*_SSE_(*H* = 0) remain zero, and both increase when *V*_G_ is larger than 13.5 V. Moreover, as *V*_G_ reaches 16 V, the contribution of **C** turns to a saturation, indicating that the thermal assisting effect is strong enough for the reversal of the spin chirality. In this view, although smaller than the contribution of **M**, the contribution of spin chirality to the SSE can be distinguished.

The role of **C** in the variation of *V*_SSE_ is further verified with **M** set to zero by zero–magnetic field cooling from 300 K, where an initial state of (**M** = 0, **C** = 0) is achieved due to a complete demagnetization (fig. S8). Therefore, *V*_SSE_(*H* = 0) equaling to zero is attained, as plotted by the black symbols in Fig. 5D. Subsequently, under applications of *V*_G_ of +16 and −16 V, *V*_SSE_(*H* = 0) is switched between negative and positive values, corresponding to additional multivalued states that can be reversibly and reproducibly written by *V*_G_ and distinguishably read via the SSE. In summary, by controlling the contributions of the spin chirality and the magnetic moment, unlimited multivalued states via a continuous variation of *V*_G_ can be demonstrated, promising great significance and potential for new types of spintronic devices.

## DISCUSSION

In summary, we report an all-electrical reading and writing of spin chirality in a noncollinear AFM insulator: The out-of-plane vector spin chirality in multiferroic TbMnO_3_ epitaxial films can be electrically detected through the SSE and switched by an electric field. Multivalued states are further achieved by controlling the thermal-assisted switching of the spin chirality by various *V*_G_. We demonstrate that, by taking advantage of the influence of the spin chirality on magnon transport, the SSE can be extended to chiral antiferromagnets as a method to characterize the switching of the spin chirality. Moreover, all-electrical reading and writing of spin chirality can be expected in other multiferroic systems such as BiFeO_3_ and the recently discovered van der Waals multiferroic NiI_2_ (*19*). Our results have applications beyond AFM spintronics, to magnonics and oxide spin-orbitronics (*34*–*41*).

## MATERIALS AND METHODS

### TbMnO_3_ growth

Epitaxial single-crystalline TbMnO_3_ films, 20 nm thick, were grown on LaAlO_3_ (110) single-crystalline substrates via pulsed laser deposition using a 248-nm KrF excimer laser at a substrate temperature of 750°C and an O_2_ pressure of 100 mtorr. High-energy optical pulses, 20 ns long, with an energy density of 1 J/cm^2^, at a repetition frequency of 3 Hz, were used with a target-substrate distance set to be 5 mm. After growth, the films were cooled down to room temperature in an O_2_ pressure of 100 torr.

### Characterization of thin films

The surface topography of the as-deposited films was characterized using an Asylum Research Cypher VRS atomic force microscope. θ-2θ X-ray diffraction scans were carried out with a Bruker D8 Discover X-ray four-circle diffractometer using Cu-Kα radiation with a tube voltage of 40 kV and a current of 40 mA.

### Device fabrication for measurements of the SSE

The as-grown TbMnO_3_ thin films were ultrasonically cleaned sequentially in acetone, ethanol, and deionized water. A positive resist (AR-P 679.03) with a thickness of ~120 nm was used for e-beam lithography, using Electra 92 as a conducting top layer to dissipate additional electrical charge. A dose of 150 μC/cm^2^ in a Raith Pioneer system was used to thereby create nanostrip patterns for nonlocal measurements of the SSE. After exposure, the Electra 92 layer was removed in water and the pattern was developed using the developer AR 600-56. Then, a 6-nm-thick Pt layer was deposited in an AJA direct current (DC) magnetron sputtering system, and the Pt nanostrips were completed via a liftoff process using acetone. Next, the electrodes were fabricated via photolithography using a positive resist (AR-P 3540T) with a thickness of 1.5 μm and developed in the developer AR 300-26. Last, film stacks consisting of Ru (5 nm)/Au (70 nm) were deposited in a scia 200 milling/deposition system and lifted off to complete the devices.

### Setup for nonlocal measurements of the SSE

Second harmonic measurements were carried out using the above nonlocal devices. The devices have Pt nanostrips that are 100 μm long, are 200 nm wide, and are spaced from each other by 400 nm. A physical property measurement system (PPMS) from Quantum Design was used to carry out the electrical measurements at variable temperatures down to 4 K and in a magnetic field of up to 90 kOe. An AC source from an SR830 lock-in amplifier with a voltage range of 0 to 4.5 V was used to generate a periodic AC in the Pt spin injector at a frequency of 13.137 Hz. The second harmonic voltage signal from the Pt spin detector was recorded by the same SR830 lock-in amplifier, thereby giving *V*_SSE_ (*27*, *42*, *43*).

### Gating process and following measurement of the SSE

For the gating process, *V*_G_ is first applied to both of the Pt strips (the spin injector and the spin detector) oriented along the *c* axis, as sketched in Fig. 3A. Considering the distribution of *V*_G_ on the Pt strips, the two terminals of the Pt strips serve as the positive and negative electrodes and form an electric field along the *c* axis, which is localized in the area near the Pt strips due to the insulating behavior of the bottom TbMnO_3_. Compared to the usual application of electric field with small leakage currents, *V*_G_ applied to the Pt strips (e.g., 16 V) generates a large current and thus a notable heating effect, which brings the temperature of the Pt strips to above the critical temperatures of the FE and AFM orderings of the TbMnO_3_ (as estimated in fig. S5, A to C). Then, *V*_G_ is set to 0 V at a rate of 0.1 V/s. During the decrease of *V*_G_, the temperature decreases simultaneously. When the temperature crosses the transition temperature of the FE orderings, the remaining *V*_G_ leads to a local electric field along the *c* axis in the area adjacent to the Pt strips, thus aligning the FE polarization together with the spin chirality. From this perspective, we define the gating process as a thermal-assisted switching of the local spin chirality around the Pt strips.

After *V*_G_ is set to zero, an AC source with an amplitude of 4.5 V is connected to one of the Pt strips (the spin injector) and *V*_SSE_ is read out from the other Pt strip (the spin detector) by measuring the second harmonic voltage via the nonlocal setup (Fig. 3A, right). The application of the AC voltage for *V*_SSE_ measurements induces a lateral temperature gradient over the whole device (fig. S5D). As the local spin chirality at the spin detector side varies, a variation of the magnon spin current occurs, resulting in a variation of *V*_SSE_. The increased temperature due to the application of the AC voltage is still lower than the FE and AFM transition temperatures, ensuring no disturbance to the FE and magnetic orderings (as estimated in fig. S5, A and B). To confirm no drifts of *V*_SSE_(*H* = 0) for different gating processes, full hysteresis loops are measured as a reference after the measurement of the *H*-dependent *V*_SSE_ during the initial magnetization.

### Quantitative estimation of the contribution to the variation of the SSE

For gating with ±*V*_G_ of the same magnitude, the electric field is opposite but the thermal effect is the same, which leads to an opposite spin chirality (±**C**) but an identical thermal-induced decrease of the magnetic moment (**M*→*M** − d**M**), respectively. Therefore, on the basis of the asymmetric variation of *V*_SSE_(*H* = 0) for +*V*_G_ and −*V*_G_, the contribution of **C** and **M** can be quantitatively estimated. The difference between the final values of *V*_SSE_(*H* = 0) set by +*V*_G_ and *−V*_G_, as denoted by *V*_SSE, +*V*_G__ and *V*_SSE, −*V*_G__, is determined by the opposite contributions of +**C** and −**C***.* Therefore, the contribution of **C** to the variation of *V*_SSE_ can be estimated by $\$|\frac{V_{\mathrm{SSE},+V_{G}}-V_{\mathrm{SSE},-V_{G}}\;}{2}|\$$. Meanwhile, the identical thermal demagnetization effect of ±*V*_G_ leads to the same variation of *V*_SSE_. Thus, the contribution of **M** can be calculated by $\$|\frac{(V_{\mathrm{SSE},+V_{G}}-V_{\mathrm{SSE},0})+(V_{\mathrm{SSE},-V_{G}}-V_{\mathrm{SSE},0})\;}{2}|\$$, where *V*_SSE,0_ stands for the initial value of *V*_SSE_(*H* = 0) set by ±*H*_set_ before the gating process. In this way, the contributions of **C** and **M** to the variation of *V*_SSE_ as a function of *V*_G_ is given out, as summarized in Fig. 5C.

### Temperature simulation

The finite-element steady-state thermal simulations were performed on a workstation with the Heat Transfer in Solids module of Comsol Multiphysics version 5.4. The cross section of a TbMnO_3_ (20 nm)/LaAlO_3_ (2000 nm) device was modeled. The bottom surface of the LaAlO_3_ layer was kept at 4 K, while the other surfaces were defined as thermal insulation. Two Pt wires were placed on top of the TbMnO_3_ layer with volume heat source $\$P_{\mathrm{heat}}=\frac{\bar{U^{2}}\cdot wt}{\rho_{e-\mathrm{Pt}}\cdot L}\cdot \frac{1}{Lwt}=\frac{\bar{U^{2}}}{\rho_{e-\mathrm{Pt}}\cdot L^{2}}\$$, where $\$\bar{U^{2}}\$$ is the mean square of applied voltage; ρ_*e*−Pt_ = 1.06 × 10^−5^ ohm·cm is the conductivity of Pt; and *L* = 100 μm, *w* = 200 nm, and *t* = 6 nm are the length, width, and thickness of each Pt wire, respectively. The density, heat conductivity, and heat capacity from Comsol Multiphysics materials library were used for Pt, while other parameters of TbMnO_3_ and LaAlO_3_ from literatures are used (*44*–*46*), as listed in Table 1.

**Table 1.** Parameters for simulation of the temperature.

Materials	Thermal conductivity (4 K) (W m^−1^ K^−1^)	Heat capacity (4 K) (J mol^−1^ K^−1^)	Density (g/cm^3^)	Thickness (nm)
Pt	885.51	0.036	21.39	6
TbMnO_3_	1.8	0.65	7.58	20
LaAlO_3_	13.0	0.04	6.42	2000
