# Supplementary material for: All-electrical reading and writing of spin chirality
Source: Sci Adv. 2022 Dec 14;8(50):eadd6984. doi: 10.1126/sciadv.add6984 (PMC11804164; doi:10.1126/sciadv.add6984)
Supplement: Supplementary file 1 — Supplementary Text Figs. S1 to S8 Table S1 [file sciadv.add6984_sm.pdf]

Science Advances
AAAS

Supplementary Materials for

All-electrical reading and writing of spin chirality

Fan Li *et al.*

Corresponding author: Fan Li, fan.li@mpi-halle.mpg.de; Stuart S. P. Parkin, stuart.parkin@mpi-halle.mpg.de

Sci. Adv. **8**, eadd6984 (2022)
DOI: 10.1126/sciadv.add6984

This PDF file includes:

Supplementary Text
Figs. S1 to S8
Table S1

Supplementary Text

Analysis of the lattice misfit between $TbMnO_3$ and various substrates

To realize the electrical detection of spin chirality in a chiral antiferromagnetic insulator, a spin chirality oriented out of plane is necessary. In the orthogonal lattice (a = 5.83 Å, b = 5.28 Å and c = 7.38 Å) of the multiferroic $TbMnO_3$, the axis of the vector spin chirality is aligned along the a-axis (*22,23*). Thus, the growth of $TbMnO_3$ (100) films with the a-axis aligned out of plane is necessary, requiring the b- and c-axis oriented in plane. We compared the lattice constant of $TbMnO_3$ with various substrates to determine the lattice misfits, as summarized in Table S1. Considering the difference between the b- and c- axis, substrates are all oriented in the direction of (110), and the two orthogonal in-plane lattice constants, i.e. (001) and ($1\bar{1}0$), are taken into the analysis. The lattice misfit is calculated as $(a_{TMO}-a_{Sub})/a_{Sub}$, where a_{TMO} and a_{sub} denote the lattice constants of $TbMnO_3$ and the substrate, respectively. Misfits larger than 15% are not listed here. As can be seen from Table S1, with the pseudocubic lattice of the substrates decreasing from 4.2 to 3.9 Å (from MgO to $LaAlO_3$), the lattice misfit between the b- and c-axis of $TbMnO_3$ and the ($1\bar{1}0$) and (001) of the substrates respectively decreases. On the other hand, for Si (110) which has a large lattice constant, there is a relatively small misfit with the b- and c-axis of $TbMnO_3$, taking into account a lattice rotation of 90 degree. Considering these lattice misfits, the two in-plane directions of $LaAlO_3$ (110), i.e. (001) and ($1\bar{1}0$) are best matched with the c- and b-axes of $TbMnO_3$, respectively. The misfit is smaller than 3%. Therefore, $LaAlO_3$ (110) was selected as the substrate, which thereby favors the epitaxial relationship: $TbMnO_3$ (100) [001] // $LaAlO_3$ (110) [001], as sketched in Fig. S1.

Table S1. Lattice misfits between $TbMnO_3$ and different substrates for two orthogonal in-plane lattices, i.e. (001) and $(1\bar{1}0)$.

			$TbMnO_3$		
			a/2 (2.91 Å)	*b*/2 (2.64 Å)	*c*/2 (3.69 Å)
$LaAlO_3$	(001)	3.79 Å	—	—	-2.6%
	$(1\bar{1}0)$	2.68 Å	8.8%	-1.5%	—
LSAT[1]	(001)	3.87 Å	—	—	-4.6%
	$(1\bar{1}0)$	2.73 Å	6.6%	-3.5%	—
$SrTiO_3$	(001)	3.91 Å	—	—	-5.5%
	$(1\bar{1}0)$	2.76 Å	5.6%	-4.4%	—
$KTaO_3$	(001)	3.98 Å	—	—	-7.3%
	$(1\bar{1}0)$	2.81 Å	3.6%	-6.2%	—
MgO	(001)	4.20 Å	—	—	-12.1%
	$(1\bar{1}0)$	2.97 Å	-1.8%	-11.1%	—
Si	(001)	5.43 Å	7.4%[2]	-2.8%[2]	—
	$(1\bar{1}0)$	3.84 Å	—	—	-3.9%

[1] LSAT is short for $(LaAlO_3)_{0.3}(Sr_2TaAlO_6)_{0.7}$.

[2] The misfit is calculated from a_{TMO} and b_{TMO} instead of $a_{TMO}/2$ and $b_{TMO}/2$, respectively.

— Misfits larger than 15% are not shown here.

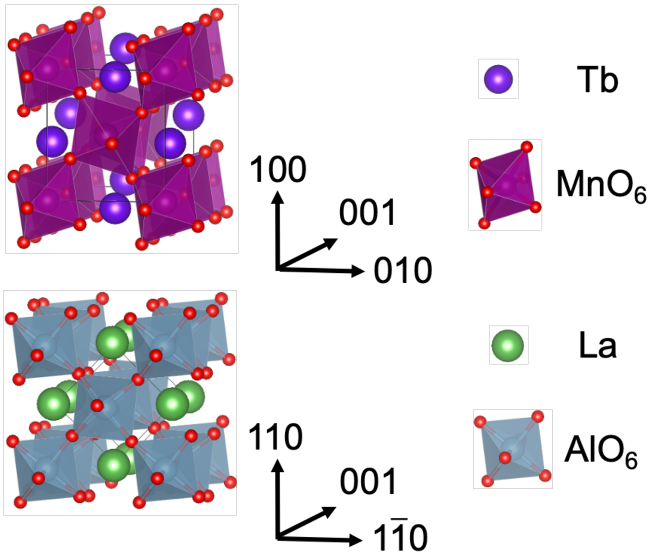

Fig. S1. Sketch of the epitaxial relationship between $TbMnO_3$ (100) and $LaAlO_3$ (110). According to the mismatch of the in-plane constant lattice, an epitaxial relationship of $TbMnO_3$ (100) [001] // $LaAlO_3$ (110) [001] is expected.

Epitaxial growth of high-quality $TbMnO_3$ (100) thin films

$TbMnO_3$ with a thickness of 20 nm is grown on $LaAlO_3$ (110). X-ray diffraction (XRD) in different directions is performed to characterize the epitaxial relationship, as shown in Fig. S2. Also shown are illustrations of the corresponding crystalline planes in $LaAlO_3$ (LAO) and $TbMnO_3$ (TMO), according to the expected epitaxial relationship of $TbMnO_3$ (100) [001] // $LaAlO_3$ (110) [001]. An epitaxial XRD peak of TMO (200) is observed near the peak of LAO (110), demonstrating that the *a*-axis (the axis of the spin chirality) of TMO is aligned out of plane. From the diffraction angle (2θ) of TMO (200) at 31.10°, the *a*-axis lattice constant of the TMO thin film is calculated to be 5.75 Å, consistent with the bulk lattice constant a_{TMO} = 5.83 Å. Furthermore, XRD peaks of TMO ($2\bar{2}0$) and (202) are observed around those of $LaAlO_3$ (002) and (111), respectively, in Fig. S2B and S2C. From the diffraction angles $2\theta_{TMO(220)}$ = 46.78° and $2\theta_{TMO(202)}$ = 39.50°, the in-plane lattice constants of TMO along the *b*- and *c*-axis are calculated to be 5.27 and 7.49 Å, respectively, which are close to the bulk values (b_{TMO} = 5.28 Å and c_{TMO} = 7.38 Å). This confirms the in-plane alignment of TMO (020) // LAO ($1\bar{1}0$) and TMO (002) // LAO (001), as depicted in the sketch in the right panel. Thus, an epitaxial relationship of $TbMnO_3$ (100) [001] // $LaAlO_3$ (110) [001] is verified, in agreement with the expectation based on the small in-plane lattice misfit between TMO and LAO.

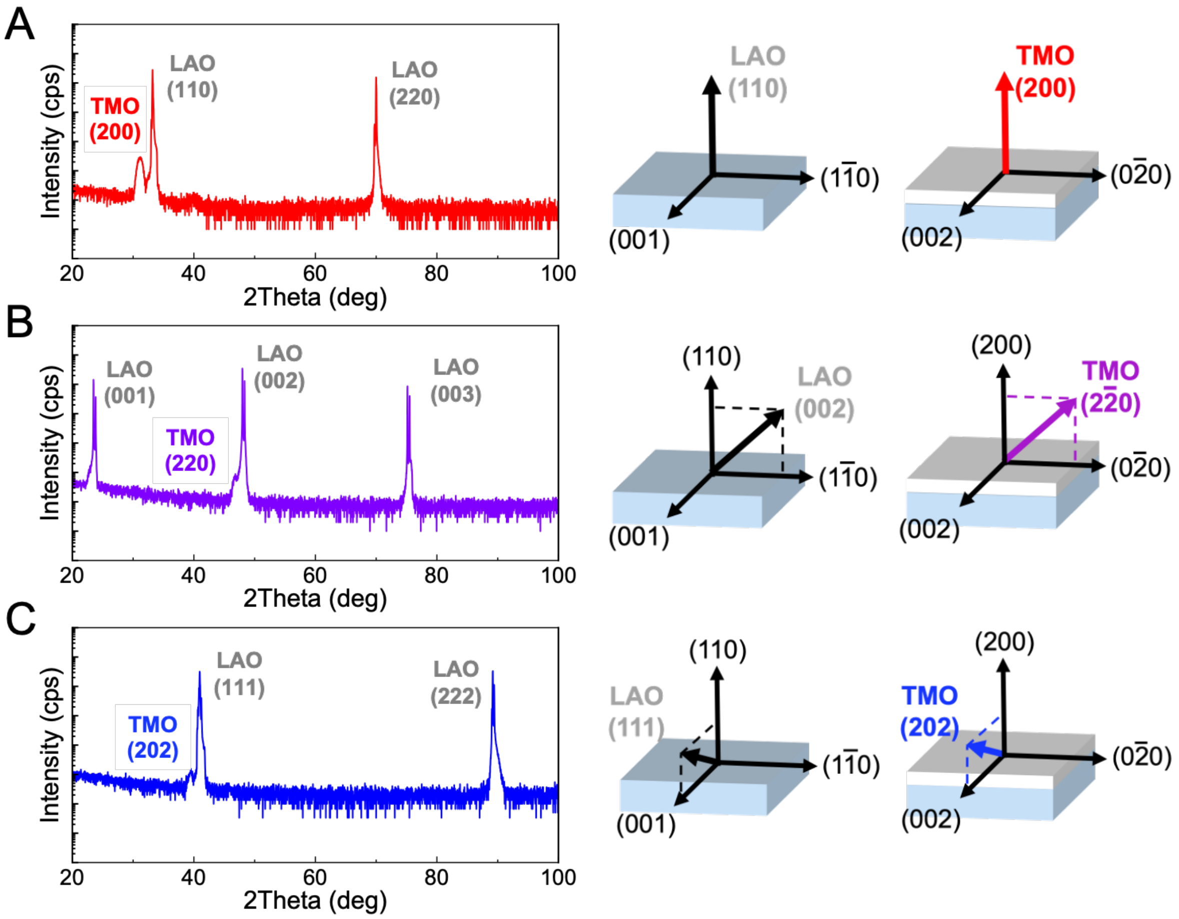

Fig. S2. X-ray diffraction and illustration of crystalline planes along different crystal orientations of the $LaAlO_3$ substrate: (**A**) LAO (110), (**B**) LAO (002) and (**C**) LAO (111).

The surface topography of the epitaxial $TbMnO_3$ film was characterized by atomic force microscopy. A smooth surface is observed, as displayed in Fig. S3. The atomic steps lead to a small roughness of 0.19 nm, demonstrating a high-quality surface of the epitaxial $TbMnO_3$ film.

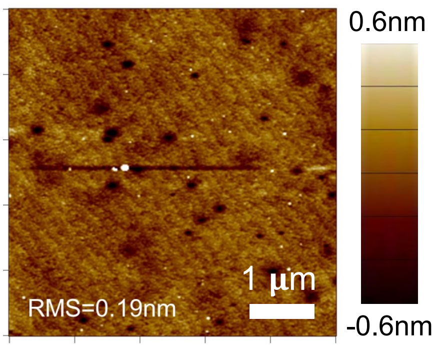

Fig. S3. Surface of $TbMnO_3$ thin film. Atomic force microscopy of 20 nm thick $TbMnO_3$ layer on $LaAlO_3$ (110) is carried out to show the smooth surface of $TbMnO_3$ thin film with a small roughness (RMS) of ~0.19 nm.

Angular dependence of the spin Seebeck effect

In order to demonstrate the angular dependence of the spin Seebeck effect, we sweep the magnetic field (*H*) in the *b*-*c* plane of $TbMnO_3$, as illustrated in Fig. S4A. θ denotes the angle between *H* and the *c*-axis. Fig. S4B plots the θ dependent V_{SSE} with different magnetic fields, which exhibits a sinusoidal behavior. Minimum and maximum values are reached at $\theta = 90°$ and 270° (H // *b*), while V_{SSE} is equal to zero at $\theta = 0°$ and 180° (*H* // *c*). This is consistent with the angular dependence of the SSE on the direction of spin polarization, where minimum and maximum values of V_{SSE} are attained when the spin polarization along the in-plane magnetic field is perpendicular to the direction of the Pt strips (*24*).

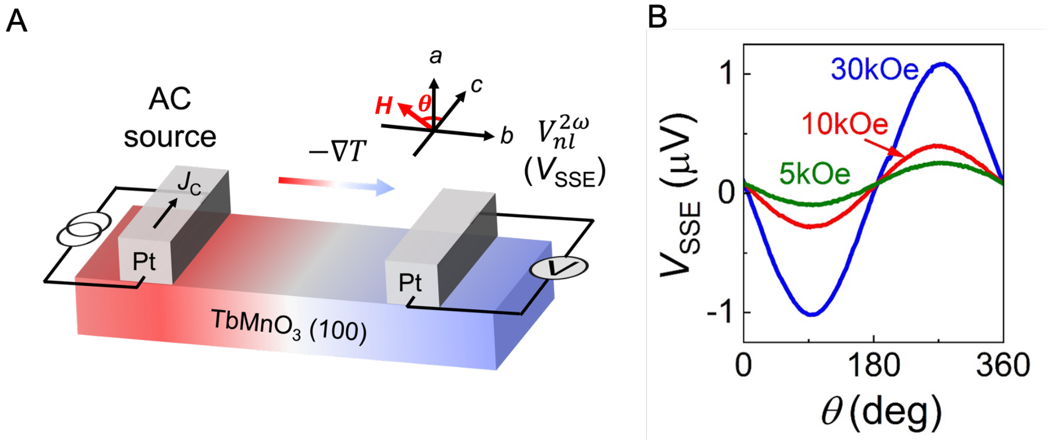

Fig. S4. Angular dependence of spin Seebeck effect. (**A**) Illustration of measurement of the angular dependence of the SSE. (**B**) Angular dependence of V_{SSE} for various magnetic fields. Maximum/minimum voltages are obtained, respectively, at 90° and 270°, i.e., *H* // *b*.

Heating effect of the Pt strips

In order to estimate the heating effect during the gating and the measurement of V_{SSE}, the temperature dependent resistance of the two parallel Pt strips is measured with a small current of 10^5 A/cm^2 to minimize the Joule heating, as plotted in Fig. S5A. Meanwhile, as V_G increases to 16 V and then decreases to 0 V with a step of 0.1 V at a rate of 0.1 V/s, the dependence of the resistance on V_G is measured, as plotted in Fig. S5B. By comparing the curves for the case with increasing and decreasing V_G (the solid and the dashed lines respectively), there is no

hysteresis. This indicates that as V_G increases/decreases to the next step, the temperature is changed to the corresponding value determined by the dependence on V_G. For gating with V_G = 16 V, according to the comparison of the temperature and gate voltage induced variation in resistance, as marked by the circular symbols in the figures, the application of 16 V on the Pt strips will induce an increase of temperature to 55 K, almost consistent with the estimated local increase of temperature to 60 K via the simulation of the temperature around the Pt strips as denoted by the black lines in Fig. S5C. The increased temperature is higher than the critical temperatures of the AFM and FE orderings. Thus, the electric-field control of spin chirality in our case can be considered as a heating-assisted switching of the spin chirality. First, the application of V_G leads to a large heating current and consequently increases the temperature of the area adjacent to the Pt strips to above the transition temperature. Subsequently, as V_G decreases at a rate of 0.1 V/s, the temperature decreases according to the dependence on V_G. When it crosses the transition temperature, there still remains an electric field (here V_G = 8 V for T = 30 K), which readily aligns the local ferroelectric polarization and finally sets the spin chirality.

In comparison, for the case of the measurement of the SSE, where an AC source with an amplitude of 4.5 V is connected to one Pt strip, the equivalent resistance is marked by the square symbols in Fig. S5A and S5B, which is calculated on the basis of an equivalent thermal power. It can be seen that, the temperature is increased to 9 K, which is still below the critical temperatures of the AFM and FE orderings. According to the simulation in Fig. S5D, a similar increase of the temperature to 6 K is estimated, together with a lateral temperature gradient covering the whole area of the devices, including the adjacent Pt strip where no current passes through.

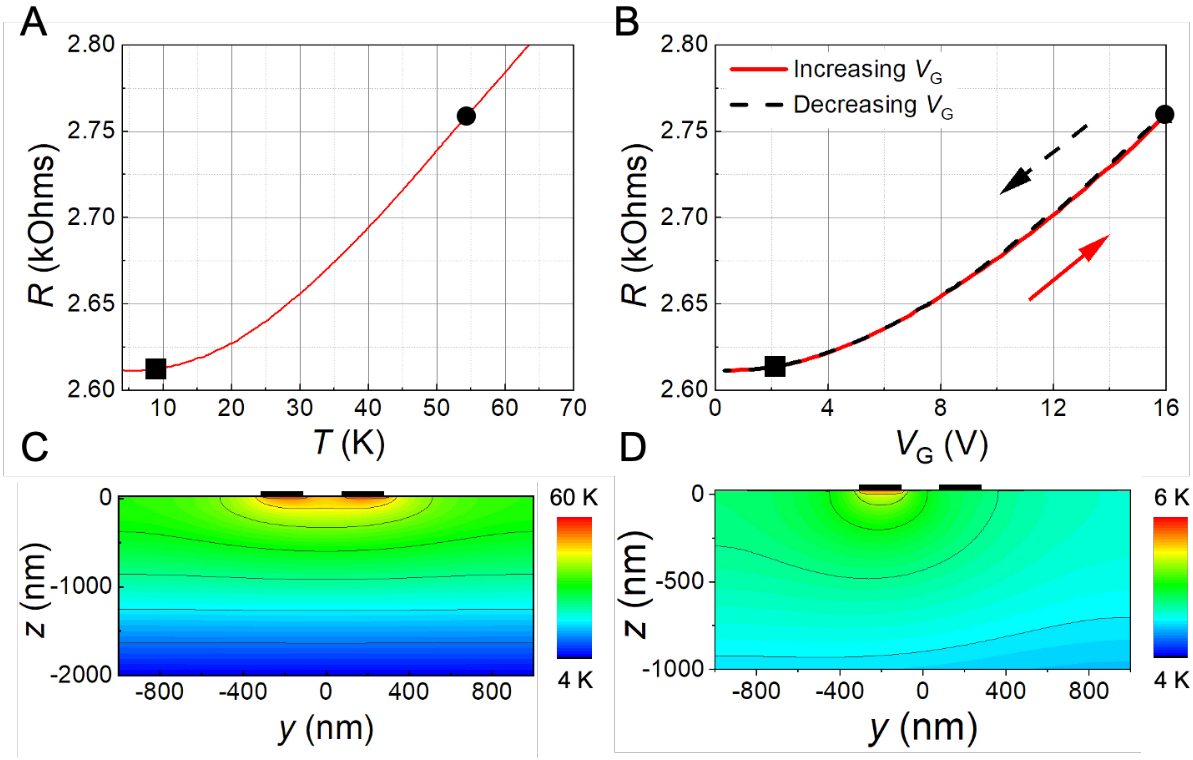

Fig. S5. Estimation of the heating effect during gating and measurements. (A) Temperature dependent and (B) gate voltage dependent resistance of two Pt strips via the warming and gating processes respectively. The circular symbols denote the resistance for the case of V_G = 16 V while the square symbols denote the resistance for the case of AC source with an amplitude of 4.5 V connected to one Pt strip, considering the equivalent thermal power. Solid and dashed lines in (B) stand for the increasing and decreasing processes of V_G during the gating. Simulation of the temperature distribution for the case: a gate voltage of 16 V is applied to the two Pt strips (C) and an AC source with an amplitude of 4.5 V is connected to one Pt strip (D). The black lines in (C) and (D) denote the positions of the Pt strips.

Background of the Spin Seebeck effect

Since the spin spirals in $TbMnO_3$ can be manipulated by a magnetic field, the spin Seebeck effect should show a response as the spin chirality is varied by the application of a magnetic field. The magnetic field-dependence of the spin Seebeck voltage (V_{SSE}) at different temperatures was measured over a large range of magnetic field that was applied along the *b*-axis (*H* // *b*). As the magnetic field increases to large values, the magnitude of V_{SSE} begins to saturate, as shown in Fig. S6. This can be ascribed to a gradual spin-flop transition occurring around 50 kOe when the magnetic field is applied along the axis of the spin spiral, i.e., the b-

axis (*31,33*). Moreover, as the temperature increases and approaches T_N (41 K), the spin chirality formed by the chiral antiferromagnetic order becomes weaker, and thereby the spin Seebeck effect disappears. Thus, the variation of the magnitude of the spin chirality in response to changes in the magnetic field and temperature induces an additional V_{SSE} response. For the measurement range used in our studies from −30 to 30 kOe, the influence of the spin-flop transition is not significant so that a linear background is taken into account in our data analysis.

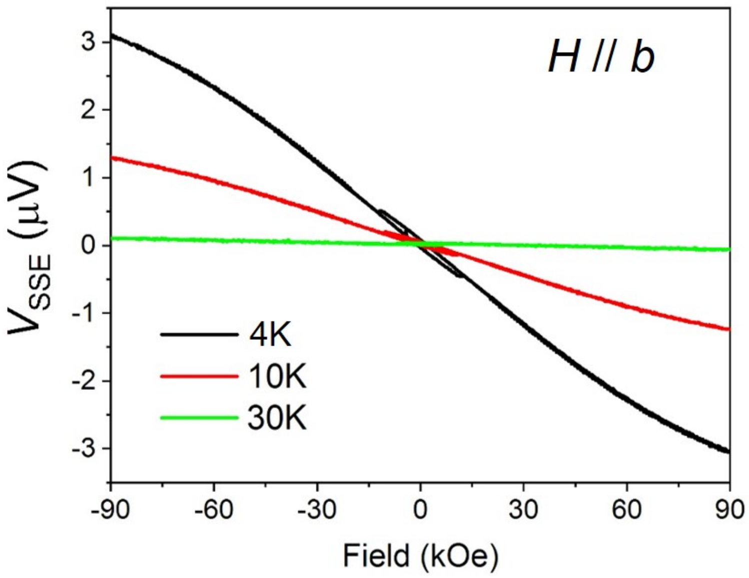

Fig. S6. **Temperature dependent spin Seebeck effect of the $TbMnO_3$ along the *b*-axis.** Magnetic field-dependent V_{SSE} over a large range of magnetic field from −90 to 90 kOe at several temperatures. The magnetic field is applied along the *b*-axis.

Zero-field spin Seebeck voltage after application of various magnetic fields and gate voltages

Since the spin chirality can be reversed via a gate voltage and manipulated by the magnetic field, application of different magnetic fields and gate voltages should cause different spin chirality states, which can then be read out as different spin Seebeck voltage (V_{SSE}) values. Figure S7 demonstrates the variation of the zero-field V_{SSE} at 4 K after applying various magnetic field (H_{set}) and gate voltage (V_G) values. V_G is applied after H_{set} is set to zero, and V_{SSE} is measured with $H_{set} = 0$ and $V_G = 0$. Following repeated settings of $\pm H_{set}$ and $\pm V_G$, four-valued

states are achieved that show a reversible and reproducible behavior. After each setting of H_{set} and V_G, V_{SSE} is measured 5 times which is used to set the error bar for each zero-field V_{SSE} state as shown in Fig. 4.

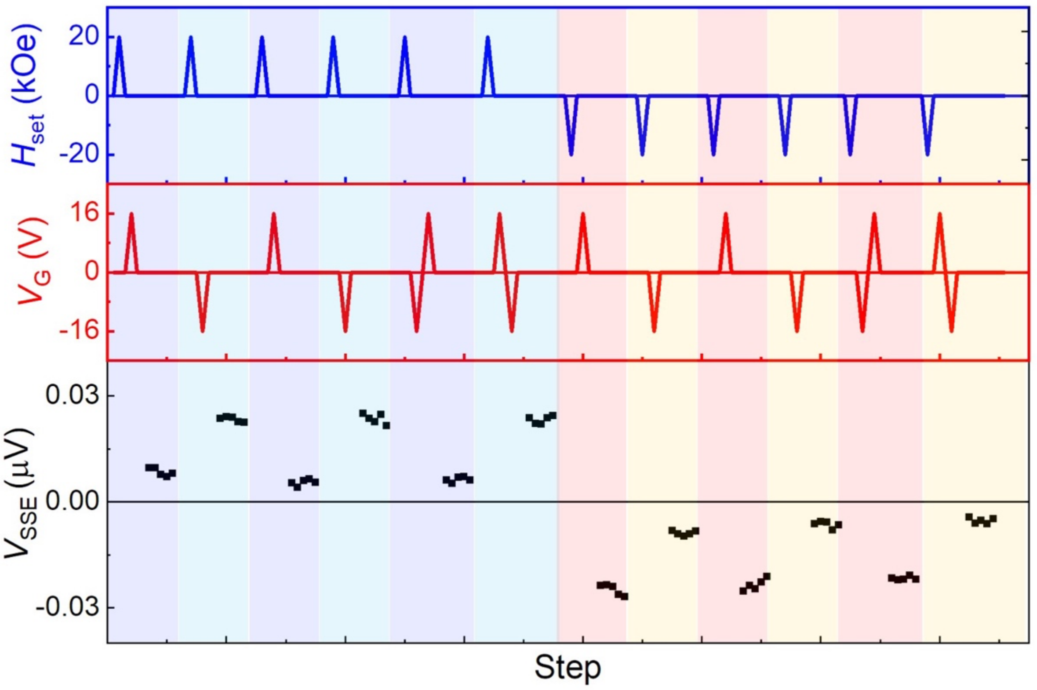

Fig. S7. **Multi-valued states of the zero-field spin Seebeck voltage.** The multi-valued V_{SSE} is obtained at 4 K via the application of different magnetic fields H_{set} and gate voltages V_G as illustrated by the upper two panels.

Zero-magnetic-field cooling of $TbMnO_3$ for zero net magnetic moment

To reveal the role of spin chirality to the spin Seebeck effect with no influence of the magnetic moment, zero-magnetic-field cooling from 300 K can be an effective method, where a complete demagnetization occurs with the net magnetic moment set to zero. A zero net magnetic moment in $TbMnO_3$ achieved by the zero-magnetic-field cooling is confirmed, according to the temperature dependent magnetization (the red line) in Fig. S8A, as compared to a small magnetic moment achieved by magnetic field-cooling with $H = 5$ kOe. In this way, a value of $V_{SSE}(H=0)$ equaling to zero is attained as shown by the dot-dashed line in Fig. S8B. Furthermore, via a subsequent gating with $V_G = +16$ and -16V, negative and positive values of

V_{SSE}(H=0) are gained respectively, verifying the role of the reversal of the spin chirality in the variation of the SSE with no influence of the net magnetic moment.

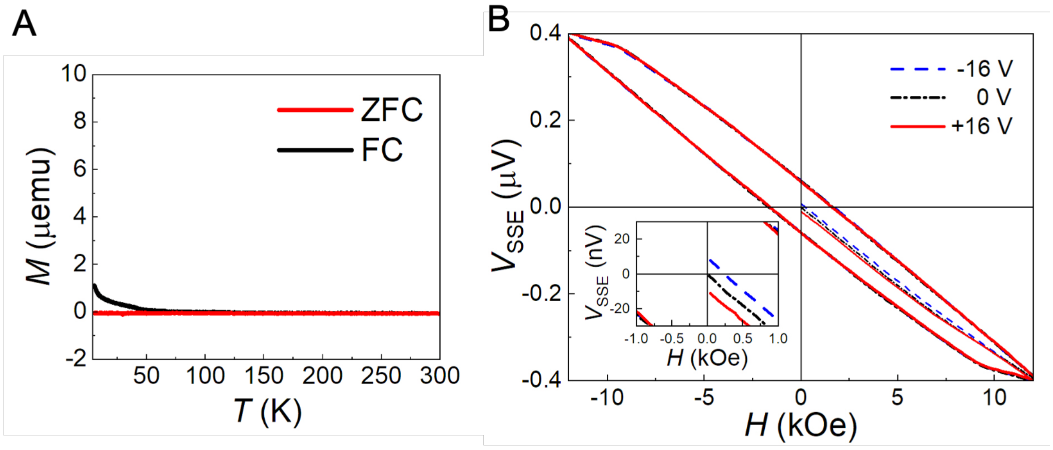

Fig. S8. Zero net magnetic moment achieved by zero-field cooling for subsequent gating. (**A**) Temperature dependent magnetization of $TbMnO_3$ thin film along b-axis. Zero magnetic field-cooling (ZFC) and magnetic field-cooling (FC) with H = 5 kOe from 300 K are carried out and the temperature dependent magnetization is measured with H = 10 Oe during the warming process. (**B**) V_{SSE} as a function of H after only ZFC (marked as 0 V, the dot-dashed line) and ZFC together with a subsequent gating with V_G = +16 and −16V (the dashed and solid lines). V_{SSE} is measured with H sweeping from 0, including the initial magnetization process followed by a full hysteresis loop. Variation of V_{SSE} (H = 0) around the origin is shown in the inset for clarity.
